# Recurrence of task-related electroencephalographic activity during post-training quiet rest and sleep

**DOI:** 10.1038/s41598-018-23590-1

**Published:** 2018-03-29

**Authors:** Michael Murphy, Robert Stickgold, Mittie Elaine Parr, Cameron Callahan, Erin J. Wamsley

**Affiliations:** 1 Harvard Medical School, Boston, MA 02115 USA; 2 McLean Hospital, Belmont, MA 02478 USA; 3 Beth-Israel Deaconess Medical Center, Boston, MA 02215 USA; 4 Furman University, Greenville, SC 29613 USA

## Abstract

Offline reactivation of task-related neural activity has been demonstrated in animals but is difficult to directly observe in humans. We sought to identify potential electroencephalographic (EEG) markers of offline memory processing in human subjects by identifying a set of characteristic EEG topographies (“microstates”) that occurred as subjects learned to navigate a virtual maze. We hypothesized that these task-related microstates would appear during post-task periods of rest and sleep. In agreement with this hypothesis, we found that one task-related microstate was increased in post-training rest and sleep compared to baseline rest, selectively for subjects who actively learned the maze, and not in subjects performing a non-learning control task. Source modeling showed that this microstate was produced by activity in temporal and parietal networks, which are known to be involved in spatial navigation. For subjects who napped after training, the increase in this task-related microstate predicted the magnitude of subsequent change in performance. Our findings demonstrate that task-related EEG patterns re-emerge during post-training rest and sleep.

## Introduction

A robust body of animal literature suggests that task-related patterns of brain activity are “replayed” during subsequent periods of quiet rest and sleep. When rodents navigate a maze, a particular hippocampal “place” cell will selectively fire when the animal is in a particular part of the maze^1^. Thus, as the animal moves through the maze there is a sequence of place cell firing that corresponds to its path through the maze. These patterns of neural firing are replayed during sleep and rest^2–4^. This hippocampal replay is associated with concomitant replay in the cerebral cortex^5^, and may be important for the transfer of information from short-term storage in the hippocampus to longer-term storage in the cortex. In rodents, replay has been observed primarily during NREM sleep (corresponding to stage N2 and N3 sleep in humans) and drops off quickly with time elapsed since sleep onset^3,5,6^.

There has been little direct evidence for an analogous process of offline memory “reactivation” in humans, in large part due to technical limitations. Widely used neuroimaging techniques such as PET and fMRI lack the spatial and temporal resolution to characterize patterns of activity at the cellular level in the same manner that these effects have been described in rodents. However, there is evidence suggesting that replay-like phenomena may occur in humans. First, multiple neuroimaging studies have shown that brain activity during periods of rest and sleep is shaped by prior learning experience^7,8^. Furthermore, in several studies the magnitude of these effects has been shown to predict subsequent memory performance^9–11^. For example, PET-defined reactivation of hippocampal activity during sleep following training on a virtual maze navigation task has been shown to predict overnight improvement on the task as has increased slow wave activity in task-related regions^9,12^. Complementing this work, cognitive studies have shown that learning a task influences the content of subsequent thoughts, mental imagery, and dreams^13,14^, which in turn can predict subsequent task improvement^15^.

In the current study, we asked whether similar experience-dependent changes in resting state brain activity might be observable using electroencephalography (EEG). Prior investigations have demonstrated that slow wave activity during sleep shows localized experience-dependent increases during N2 and N2/N3 combined sleep^12,16^. Furthermore, multivariate pattern classification has been used to classify sleep EEG data based on the content of prior visual experience^17^. However, EEG has not previously been used to assess the reactivation of task-related brain activity during post-learning rest. In this study, we examined memory processing related to learning a complex spatial navigation task. Previous work with this task suggested an important role for N2 sleep^18^. Because of this, and in combination with evidence that memory reactivation is strongest immediately following experience, we hypothesized that experience-dependent changes in resting state brain activity would be largest during quiet rest and N2 sleep immediately following training on the task.

We applied the technique of “microstate analysis” to this question, identifying spatial patterns of scalp EEG activity associated with training on a virtual maze navigation task, and tracking the persistence of these EEG patterns across subsequent periods of rest and sleep. Microstate analysis offers the unique ability to simplify millions of samples of time-series EEG data to a small number of repeatedly recurring scalp voltage topographies called microstates^19,20^. This analysis focuses on the *spatial pattern* of voltage across the scalp, as opposed to traditional spectral analysis, which focuses on the frequency of oscillations. Microstates have been proposed to reflect the semi-stable integrated activity of large-scale functional networks which give rise to sequential episodes of consciousness^21^. We hypothesized and then confirmed that task-related EEG topographies (microstates) recur during subsequent periods of quiet rest and sleep.

## Materials and Methods

### Subjects

Subjects (n = 78, 33 women, ages 18–30) were English-speaking students recruited from local colleges and universities using online advertisements. Informed consent was obtained from all subjects. Subjects agreed to keep a regular sleep schedule for three nights prior to the study and obtain a minimum of 6 hours of sleep on average during those three nights, as documented by a retrospective sleep log completed at the study visit. Subjects also agreed not to consume caffeine after 10 am on the day of the study. We excluded individuals who reported a diagnosis of sleep or psychiatric disorders and individuals who took medications known to interfere with normal sleep or cognition. We also excluded individuals who had already participated in a study that used the Virtual Maze Task. In previous studies using this task, we found that individuals with only very limited experience playing spatial navigation video games did not reliably learn the task in the time allotted^18^. Therefore, in this study we excluded individuals who reported playing 3D style video games less than once a year.

Seven subjects enrolled but failed to complete the study. Data from fifteen subjects was not analyzed because they either fell asleep during quiet rest periods or did not nap during nap periods (described below). Data from fifteen additional subjects was not analyzed due to persistent EEG artifact (defined as more than 5 bad electrodes that could not be corrected with spline interpolation). Ultimately, data from 41 subjects (18 female, mean age = 20.6 years, ±1.5 years) were analyzed for this study.

### Task

**Figure 1:**
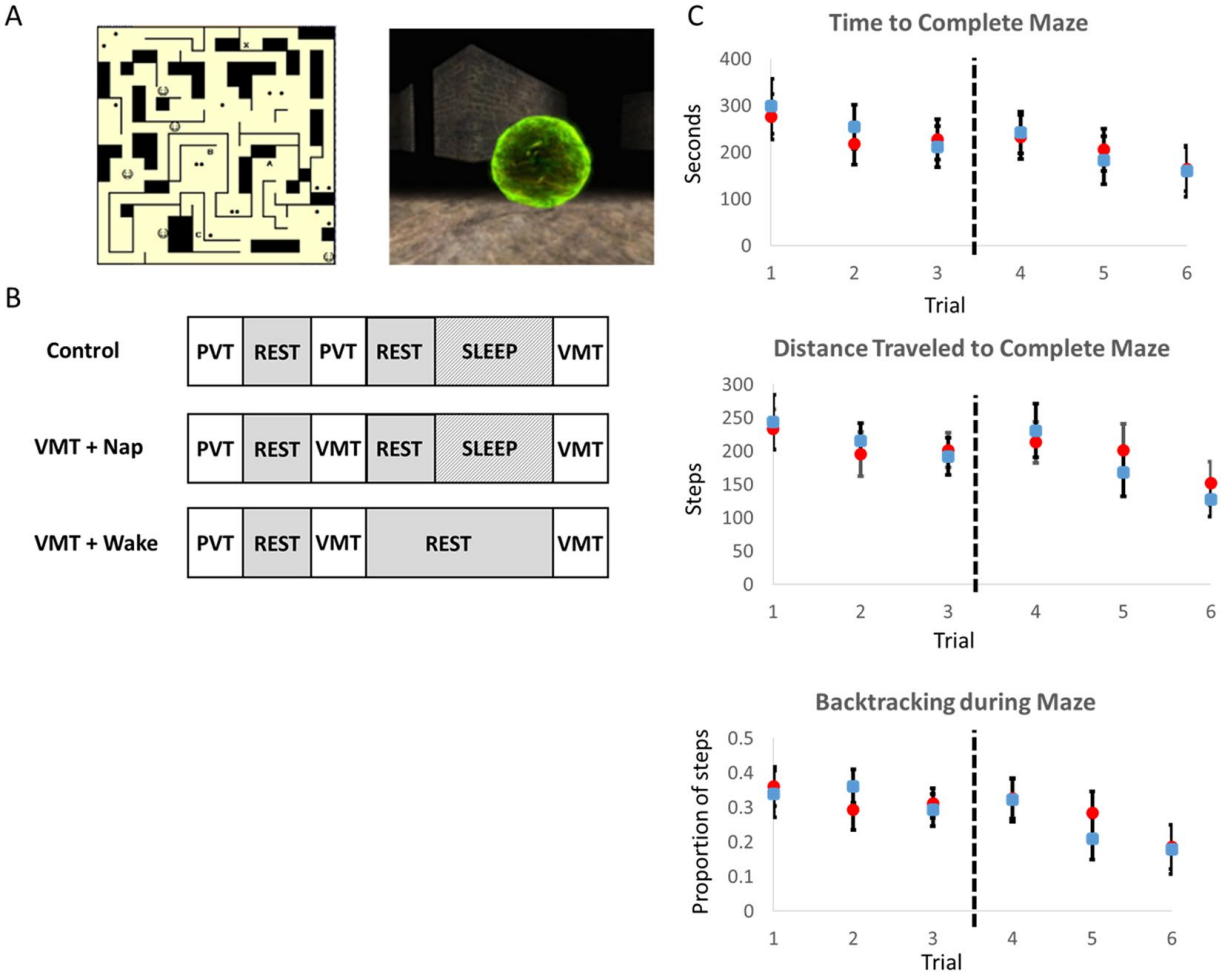
(**A**) Virtual maze task. The left panel shows the layout of the virtual maze. (Subjects never see this view). The right panel shows one view from within the maze, as it appears to subjects. (**B)** Experimental design. All subjects begin by performing the psychomotor vigilance task (PVT) as a baseline control at 12 pm. Then, after a seven minute period of quiet rest, one group of subjects performs the PVT again, and the remaining subjects train on the virtual maze task (VMT). Following the VMT or PVT, there is another seven minute rest period, after which half of subjects who completed the VMT training and all of the PVT control subjects are allowed a 90 minute nap opportunity. At 4:30 pm, two hours after the completion of this nap/rest period, all subjects are tested on the VMT. (**C)** Task performance. Three bar plots showing average time to navigate the maze, average amount of distance traveled before reaching the exit, and proportion of steps that were back-tracking for subjects who napped (red circles) and wake subjects (blue circles).

All study procedures were approved by the Committee on Clinical Investigations which is the institutional review board for the Beth Israel Deaconess Medical Center. All experiments were performed in accordance with relevant guidelines and regulations. The Virtual Maze Task (VMT) is a spatial navigation task in which subjects use a computer keypad to navigate through a complex maze environment (Fig. 1A). During a five-minute exploration period, subjects are placed at the maze exit and instructed to learn the layout of the maze as well as possible, so they will be able to navigate to the maze exit as quickly as possible during subsequent tests. There are then three test trials, each beginning from a different point in the maze equidistant from the exit (order of starting points counterbalanced across subjects). If subjects fail to reach the exit within 10 minutes, the trial is ended and a new trial is begun. At retest, three additional trials are conducted. Performance on each trial is assessed in terms of both the time taken and distance traveled to reach the exit. We also calculated the number of unique positions encountered while navigating the maze and the amount of backtracking (1-(unique positions/distance traveled))^22^. Improvement is calculated as the change in performance from the last training trial to mean performance at test. Therefore, a positive change from training to test reflects deterioration and a negative change reflects improvement.

Instead of performing the maze task, subjects in the non-learning Control group completed the Psychomotor Vigilance Task (PVT^23^), a simple measure of vigilance in which participants are asked to press a key whenever a red dot appears on the computer screen. The PVT measures reaction-time and is not susceptible to learning effects^24^. The number of PVT trials completed was yoked to the duration of maze training in the learning group on a subject-by-subject basis in order to equate on-task time across groups.

### Procedure

The study design is illustrated in Fig. 1B. Subjects were divided into three groups: a “Nap” group, “Wake” group, and “Control” group. All three groups began by performing the Psychomotor Vigilance Task (PVT). This ensured that all subjects were in a similar state at the start of the experiment. Following the PVT, there was a seven minute period of baseline eyes-closed quiet rest for all subjects. The Nap group (n = 14, mean age = 21.2, ±1.4 years s.e.m.) then trained on the Virtual Maze Task (VMT) followed by a second period of quiet rest and then a 90-min nap opportunity. Following the nap period, these subjects were retested on the VMT. The Wake group (n = 13, mean age = 20.3, ±1.7 years) trained on the VMT as above, but did not nap following the second rest period and instead watched preselected PG-rated movies and ate lunch. The Control group (n = 14, mean age = 20.2 years, ±1.4 years) did not train on the VMT, but instead performed additional PVT trials as described above. Control subjects also had a 90 minute nap opportunity after which they were then trained and tested on the VMT.

The PVT was chosen as the control task because it has a minimal learning curve with skill acquisition complete after 1–3 trials^23^. Therefore, any PVT learning occurs early in the initial PVT block, which all subjects perform, with no further learning during subsequent (Control only) PVT blocks.

### Recordings and Analysis

Throughout the duration of the study, 60 channels of EEG were recorded using a 64-channel Grass-Telefactor Aura system, sampling at 400 Hz. EEG data were preprocessed by manual artifact rejection, 0.5–40 Hz band-pass filtering, spline interpolation of bad channels (average of 1.5 channels per subject), and average referencing. Sleep was scored according to the standard criteria of the American Academy of Sleep Medicine^25^. Microstate analysis focused on stage N2 sleep because in previous work we showed that post-nap improvement in the VMT was correlated with EEG delta power during N2 sleep^18^. In addition, the vast majority memory reactivation studies in rodent models have described replay effects in NREM, rather than REM sleep^26^.

Segments of data with ocular artifact were identified by visual inspection of the data. These segments were removed. Spectral analysis was performed in MATLAB using Welch’s method with a Hanning window of 4 seconds with 50% overlap (Mathworks, Natick, MA). Linear regression calculations were also performed in MATLAB.

SnPM suprathreshold cluster tests were used to compare the scalp topography across experimental group, behavioral state, and frequency band^27^. In this method, study data are randomly assigned to group across thousands of iterations. For each iteration, between-group t-tests are generated for each channel and compared to a predetermined threshold value. The number of contiguous channels that exceed this threshold is the cluster size for that iteration. This is repeated thousands of times to create a null-distribution of cluster sizes. The cluster size of the correctly grouped data is then compared against this distribution.

### Microstate Analysis

Microstate analyses model spatial patterns in the EEG over an extended recording period as a series of transitions between a small number of topographies that explain the majority of the spatial variability. This is done by applying a simple cluster analysis to a time series of scalp topographies. Microstate topographies are derived from repeated unbiased randomly-seeded cluster analyses of the EEG recordings and therefore are not dependent on any neurobiological assumptions^19,28^. We extracted EEG microstates from task data using widely employed methods, implemented in the Cartool software by Denis Brunet (brainmapping.unige.ch/cartool). We downsampled our data by selecting time points corresponding to peak global field power (GFP) because previous work has demonstrated that microstate topography is stable around time points corresponding to peaks in the GFP function and that transitions between microstates selectively occur at time points corresponding to local GFP minima^20,29^.

This data was then run through a k-means analysis (where k ranged from 1 to 10) to extract potential microstates repeated 300 times. We used the Krzanowski-Lai criterion to determine the number of microstates both because this method is also widely used in the literature and also because it provides an unbiased estimate^30–32^.

Once we characterized the EEG microstates during maze training, we determined how well these topographies matched the EEG data during pre-training rest, post-training rest and N2 sleep by calculating the Global Explained Variance (GEV). GEV is calculated by measuring how closely the EEG topography at each time point matches one of the maze training microstates. In this way, GEV represents the amount of topographic variance in the rest and sleep EEG recordings that can be accounted for by the microstates that were present during maze training^29^. Thus, the higher the GEV, the more the rest and sleep EEG “match” the maze-training microstates.

We hypothesized that task-related microstates would explain more of the signal (higher GEV) in post-training rest and sleep, in comparison to pre-training rest. Thus, throughout the results, GEV is presented as a measure of the degree to which the EEG topography during rest and sleep matches the set of microstates present during maze training. Statistical non-parametric mapping (SnPM) map-wise tests were used to identify statistically significant changes in microstate global explained variance^27^. In these analyses, the data from each subject consisted of the list of GEVs for all of the microstates and the permutations were performed on the lists of GEVs.

### Source modeling

Source modeling analysis was performed using the LORETA-KEY software, using a three-shell spherical head model derived from a magnetic resonance image of an individual whose head closely approximates the Montreal Neurological Institute head (“Colin 27”). A standard, co-registered set of electrode positions was used for the construction of a forward model. Standardized low resolution electromagnetic topography (sLORETA) was used to model cortical current sources^33^.

## Results

### EEG Microstates

Analysis of EEG collected during VMT training in the Nap group produced six microstate topographies, which together explained 71% of the topographic variance in the EEG signal during training. We then fit this set of training microstates to the EEG during baseline rest, post-training rest, and subsequent nap N2 sleep. There was no significant difference across behavioral states in total GEV explained by the entire set of 6 training microstates (72% in baseline pre-training rest, 73% in post-training rest, and 75% in N2 sleep).

**Figure 2:**
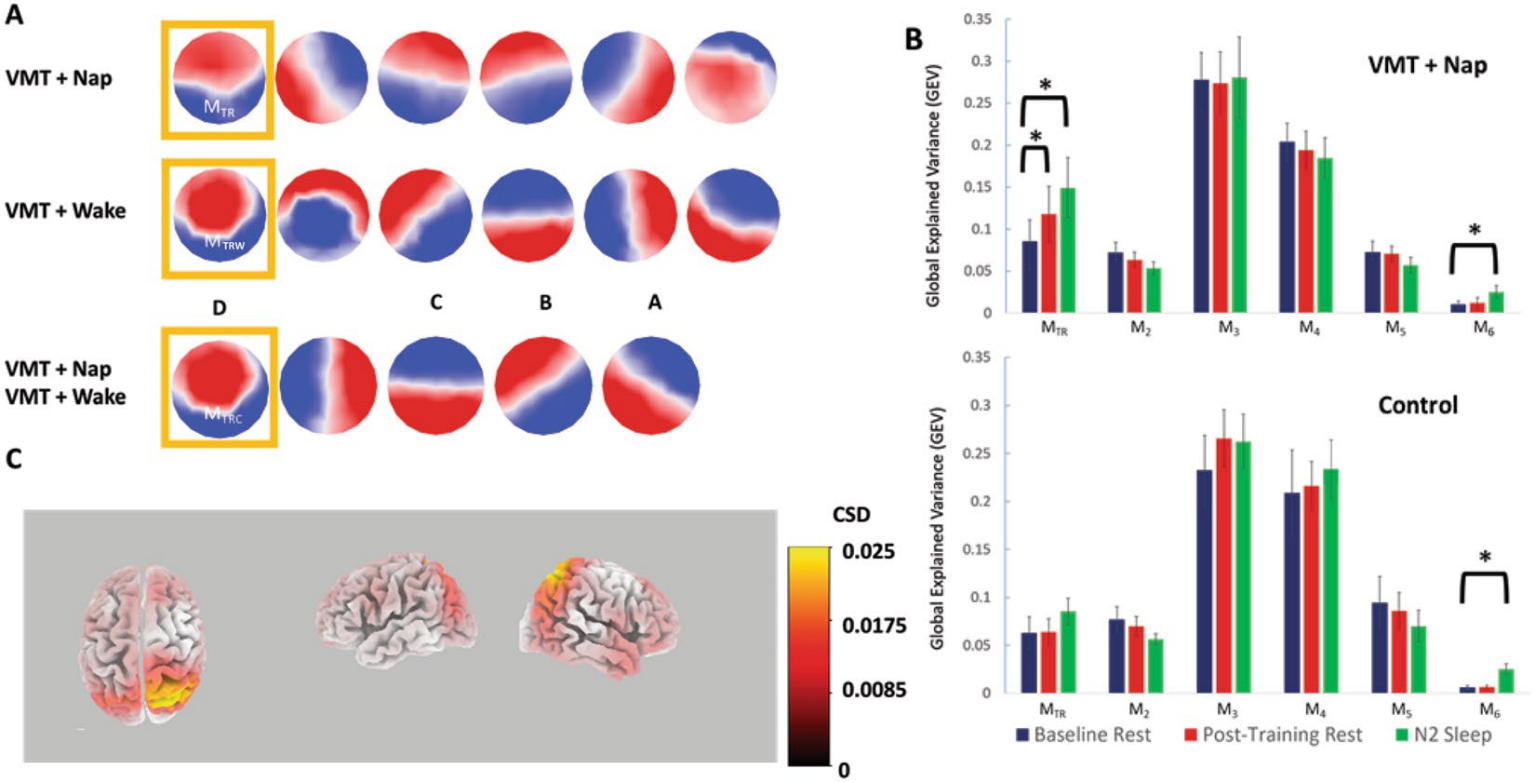
(**A)** VMT microstates. Topographic maps (red positive, blue negative) of the microstates derived from VMT training data in the Nap group, Wake group, and both groups combined. Six microstates were present in the EEG during VMT training for both Nap and Wake groups. In each analysis, one microstate (gold box) showed increased GEV in post-training rest compared to pre-training rest. In the analysis pooling all subjects who performed the VMT, five microstates were identified during VMT training. This set of microstates contains the canonical four microstates^58^, labeled here as A–D. (**B)** Fitting VMT microstates. In order to assess the presence of VMT-training microstates in rest and sleep, the six training microstates derived from the Nap group were fit to baseline rest, post-training rest, and subsequent N2 sleep in both the Nap (top) and Control (bottom) groups. Global explained variance (GEV) during baseline rest, post-training rest and N2 sleep is plotted for each training microstate (error bars: ± SEM; *SnPM p < 0.01). (**C**) Source modeling of M_TRC_. Standardized current density (calculated using sLORETA) for the M_TRC_ microstate, thresholded at 0.003). Note activation in the superior parietal lobule.

In fitting these task-related microstates to baseline rest, post-training rest and sleep, we observed that one state became significantly more prevalent during both rest and sleep after training, in comparison to baseline rest. This training-related microstate (Microstate M_TR_ [training-rest]; see gold box in Fig. 2A) showed a statistically significant increase in GEV from baseline rest to post-training rest for both raw (SnPM, p = 0.006, Cohen’s d = 0.30) and percentage change (38% increase; t-test, p = 0.001). M_TR_ increased even more in subsequent N2 sleep (SnPM, p = 0.0009, Cohen’s d = 0.24, 56% increase) (Fig. 2B, top). The increase in M_TR_ from pre-training to post-training rest also was strongly correlated with how much M_TR_ occurred during training (r_14_ = 0.80, p = 0.0006).

Importantly, this post-training increase in M_TR_ was task-specific; when this same set of microstates was fit to data from the Control group, none of the microstates showed a statistically significant difference in GEV between baseline and post-training rest (SnPM, minimum p = 0.93) or between post-training rest and N2 sleep (SnPM, minimum p = 0.06) (Fig. 2B, bottom). The selective increase of M_TR_ following maze training is substantiated by significant group (Nap vs Control) x time (pre vs. post) ANOVA interaction effects for M_TR_’s increase in both post-training rest (F_1,26_ = 4.42, p = 0.045) and subsequent N2 sleep (F_1,26_ = 6.06, p = 0.021).

As a control, we then repeated this analysis, deriving microstates from the control PVT task. None of the microstate topographies present during the PVT increased during post training rest or sleep, in either the Nap or Control groups.

In contrast, the Wake group, who also trained on the VMT, showed a strongly similar training-related microstate that increased during post-training rest. We derived VMT microstates using data from the Wake group. As in the Nap analysis, we found six microstates in the Wake group VMT training data. One microstate, M_TRW,_ strongly resembled M_TR_ (Pearson’s r = 0.85 between microstate map voltage topographies). M_TRW_ also increased significantly from pre- to post-training rest (70% increase, t-test, p = 0.033). The increase in M_TRW_ from pre-training to post-training rest also was strongly correlated with how much M_TRW_ increased during training (r_13_ = 0.76, p = 0.003).

We hypothesized that M_TR_ and M_TRW_ were capturing similar VMT-related brain activity, and that therefore these microstates would show post training increases in quiet rest even when fit to the other maze-trained group. As expected, we found that M_TR_ increased significantly following training in the Wake group (50% increase, one-sided t-test p = 0.049) and M_TRW_ increased significantly following training in the Nap group (107% increase, one-sided t-test p = 0.037).

Although our primary approach was to describe maze-related microstates separately for each group of subjects (“M_TR_” in the Nap group and “M_TRW_” in the wake group), our presumption is that these group-specific microstates reflect the same underlying brain activity in both groups. This presumption is supported by the topographic similarity M_TR_ and M_TRW,_ and the fact that both show a similar increase following maze training. Thus, in order to maximize the reliability of the microstate model, we also calculated training-related microstates in the combined Nap + Wake sample, taking advantage of the increased sample size offered by this approach. The resulting set of microstates included one, M_TRC_, that closely resembled both M_TR_ and M_TRW_ (Fig. 2A) (Pearson’s r across 60 EEG channels = 0.87 and 0.99 respectively). M_TRC_ increased in post training rest for the pooled groups (71% GEV increase, t-test, p = 0.0001). M_TRC_ also increased during post-training rest in the Nap group and the Wake group when they were analyzed independently (Nap: 77% increase, t-test, p = 0.024; Wake: 65.0% increase, t-test, p = 0.032).

A second VMT-derived microstate, which accounted for the smallest portion of the variance in every state, also increased in N2 sleep but not in post-training rest. However, this increase was seen for both the VMT and Control groups (t-test, p = 0.004 and p = 001 respectively; Fig. 2B, Microstate 6) and thus was not learning specific.

### Microstate Topography

Many previous studies of EEG microstates have reported four canonical microstates during rest and task performance^20,34–39^. In our task data, we found more than four microstates for both the VMT and control tasks. This may be related to the nature of the complex cognitive tasks that subjects were performing, or alternatively to the high-density EEG net that we used, or to the method by which we selected the number of microstates. Nonetheless, the set of microstates that we obtained from the maze task included all four canonical microstates (Fig. 2C). M_TR_, the microstate that showed increased GEV following the VMT, closely resembles M_D_, one of the four canonical microstates originally reported by Lehmann *et al*.^37^.

We used source modeling (sLORETA) to extrapolate cortical activity that was likely to be related to microstate M_TRC_. This microstate was associated with maximal cortical activity at the right superior parietal cortex (Fig. 2C).

### EEG alpha and beta power increase after task performance

**Figure 3:**
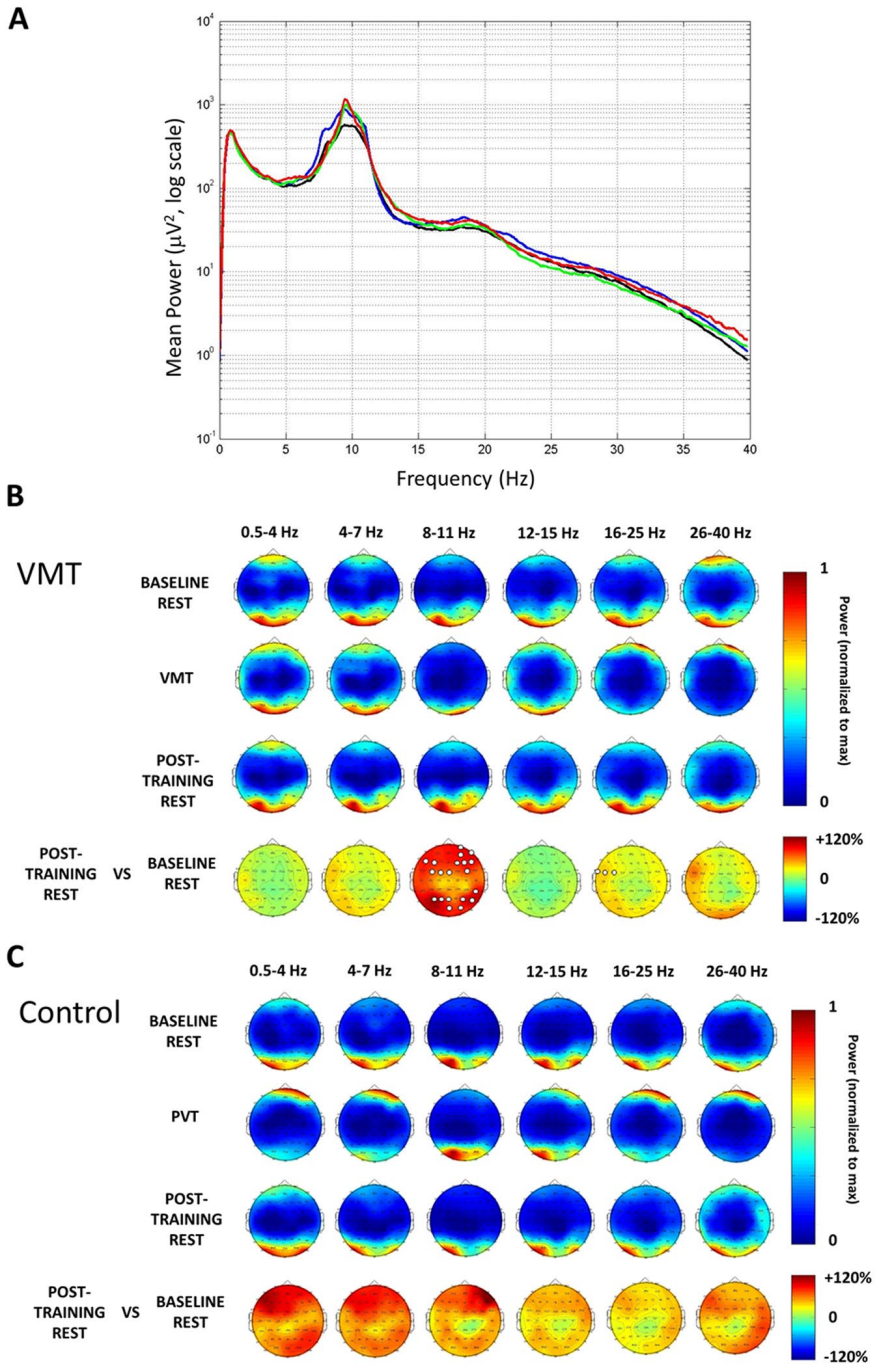
EEG power analyses. (**A**) Rest period power spectra. Power spectra were averaged over pre- and post-training rest periods; red = Control pre-training rest, blue = Control post-training rest; black = Nap pre-training rest, green = Nap post-training rest. There were no statistically significant differences between groups in any frequency band. (**B**) Topographic EEG maps of average power per channel per frequency band, normalized to the maximum power within each map for the combined Nap and Wake groups. White circles are a cluster of channels that showed an increase in resting alpha (8–11 Hz) or beta (16–25 Hz) power during post-training rest compared to pretraining rest (SnPM, p < 0.05). (**C**) Topographic EEG maps of average power per channel per frequency band, normalized to the maximum power within each map for Control subjects.

The mean power spectrum averaged across channels was grossly similar across conditions (Fig. 3A), and a one-way ANOVA did not show any statistically significant group differences in mean delta (0.5–4), theta (4–7), alpha (8–11 Hz), sigma (12–15), beta (16–25), or gamma (26–40 Hz) power when averaged across all channels (F_3,78_ = 0.02, 0.1, 0.4, 0.5, 0.3, 0.5, respectively; all p > 0.65).

However, this does not eliminate the possibility of localized differences in the EEG power. We therefore compared the scalp topography in each frequency band during the quiet rest periods, as well as during the virtual maze (Fig. 3B) and control (Fig. 3C) tasks. In Control subjects, we found no significant difference in any EEG channel in any of the six frequency bands between the pre- and post-task rest periods. We also found no significant differences in pre-training rest data between the experimental groups (not shown).

However, in subjects who completed the virtual maze, we found two disjoint clusters of electrodes that showed increased alpha power during quiet rest following training, an increase not seen in in Control participants (SnPM suprathreshold cluster test, p < 0.05; Fig. 3B). We also found a smaller set of left frontal electrodes that showed increased beta power during quiet rest following training again only in the maze task subjects and not controls (SnPM suprathreshold cluster test, p < 0.05).

### Nap structure

During the nap, subjects spent 8.8 ± 5.4 minutes in N1 sleep, 45.2 ± 19.1 minutes in N2 sleep, 17.8 ± 12.1 minutes in N3 sleep, and 10.6 ± 9.0 minutes in REM sleep. There were no statistically significant correlations between sleep architecture during the nap and subsequent performance or improvement in the VMT.

### Microstates, but not EEG power, correlate with post-sleep changes in task performance

Over the course of the experiment, subjects who trained on the VMT tended to improve in performance, for time required to reach the exit, distance traveled to the exit, and the amount of backtracking in their path (Fig. 1C). A repeated measures ANOVA revealed significant improvement in maze completion time across the 6 training and test trials (linear polynomial contrast: F_1,25_ = 11.96, p = 0.002). Although previous work using this task has demonstrated post-training nap mediated improvement in task performance^15^, here we found no differences in performance improvement between Nap and Wake subjects (p > 0.85 in t-tests for time, distance, and backtracking). This may have been due to part to sizable between-subject differences in improvement (Fig. 3).

**Figure 4:**
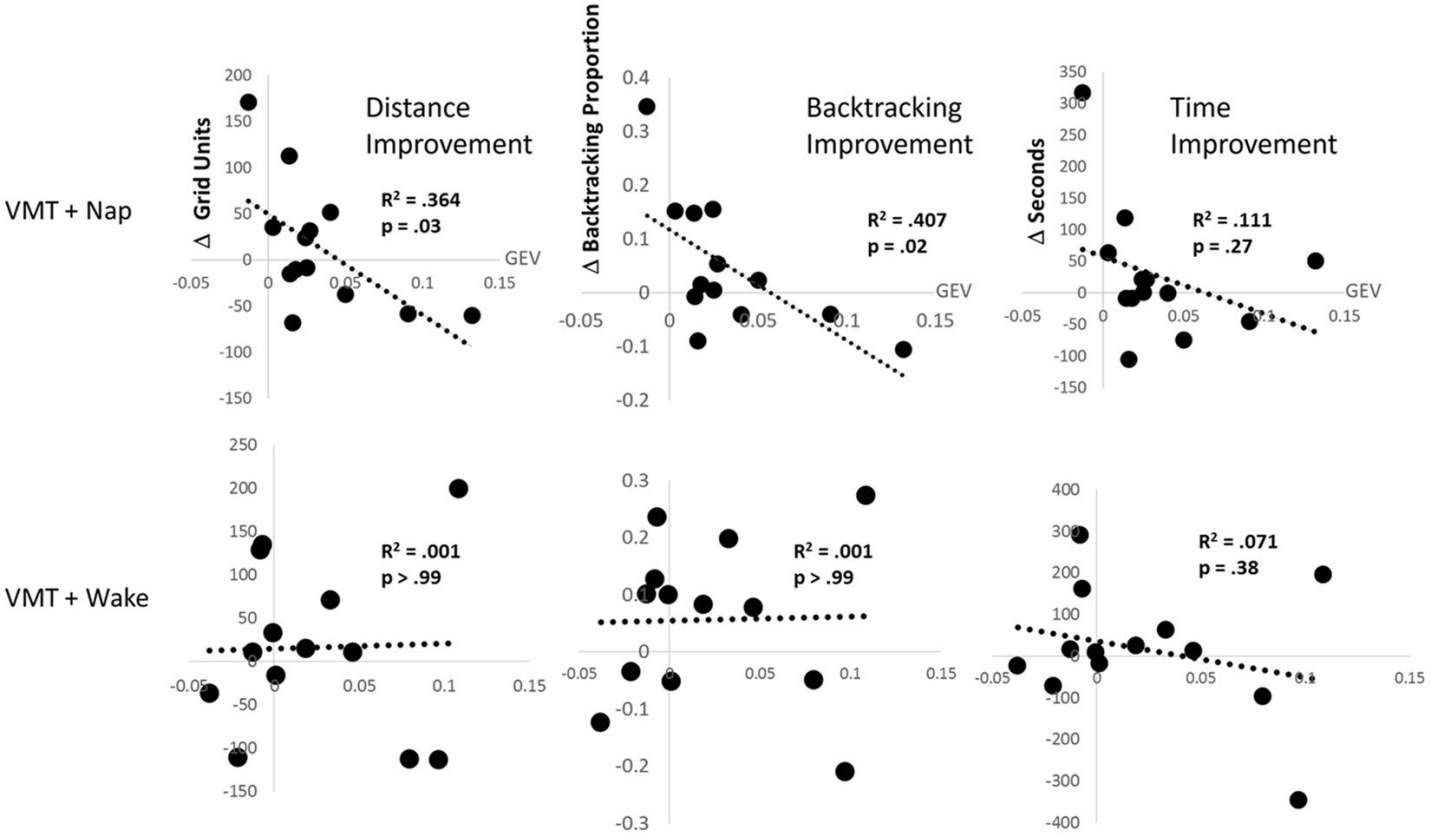
Correlations between increased post-training microstate GEV and subsequent change in VMT performance. Performance change (calculated as the change in performance from the last training trial to mean performance at test) is plotted for total distance travelled (left), backtracking (middle) and time to reach exit (right) against the change in M_TR_ GEV for the VMT + Nap group (top) and the MVT + Wake group (bottom).

Because previous work has shown that sleep EEG features may be correlated with memory even when there is not a clear overall impact of sleep on performance^40^, we asked whether the persistence of task-related EEG microstates correlated with task performance. Indeed, we found that the degree to which task-related microstate M_TR_ increased during post-training rest predicted post-nap improvement, although the correlation was negative. During retest, Nap subjects who had greater increases in M_TR_ GEV during rest covered more distance in the maze and backtracked more often (r_12_ = −0.60, p = 0.04 and r_12_ = −0.64, p = 0.02, respectively, Fig. 3, top). Given the correlation between these outcome measures, we corrected for multiple comparisons using the Dubey Armitage-Parmar method, which resulted in adjusted p values of p_adj_ = 0.047 and p_adj_ = 0.03 respectively^41^. There were no correlations between M_TR_ and baseline training performance (r_12_ = 0.19, p = 0.54; r_12_ = 0.21, p = 0.50; r_12_ = 0.29, p = 0.34 for time, distance and backtracking respectively, Fig. 4, bottom). Correlations between the M_TR_ GEV increase in N2 sleep and task improvement failed to reach significance (r_12_ = −0.36, p = 0.22; r_12_ = −0.50, p = 0.08; r_12_ = −0.47, p = 0.10; for time, distance traveled, and backtracking respectively). Wake subjects showed no improvement correlations with M_TRW_ or M_TRC_ increases_,_ regardless of whether we looked at raw changes in GEV or percent changes (r_12_ = 0.02 for both; Fig. 4).

A number of control analyses confirmed that this relationship between microstates and memory performance was specific to M_TR_ and memory improvement after sleep. Wake subjects showed no hint of improvement correlations with M_TRW_ or M_TRC_ increases_,_ regardless of whether we looked at raw changes in GEV or percent changes (r_12_ = 0.02 for both; Fig. 4). We found no correlation between total GEV from the complete set of VMT microstates in the baseline rest period and any measure of performance. Change in total microstate GEV for the complete set from baseline rest to post-training rest was also not associated with VMT performance or improvement on retest. We found no significant relationships between total microstate GEV in post-training rest or N2 sleep and any measure of task performance during training or re-test. In addition, there were no significant correlations between alpha or beta EEG power changes and learning improvement post-nap. Finally, we detected no significant correlations between the alpha and beta EEG power changes and M_TR_ GEV changes (Pearson’s r = 0.22, p = 0.45 for alpha and Pearson’s r = −0.17, p = 0.56 for beta).

## Discussion

These data clearly demonstrate that an EEG topography observed during spatial learning is spontaneously present during subsequent periods of quiet rest and sleep. To our knowledge, this is the first demonstration of a task-specific scalp EEG pattern being spontaneously expressed during post-learning rest. Interestingly, this microstate topography is associated with activity in regions of parietal cortex known to support spatial processing^42,43^. However, contrary to our expectations, the presence of this microstate during rest was *negatively* correlated with subsequent post-sleep improvement on this spatial memory task. As such, the interpretation of exactly what might be represented by the reappearance of this scalp EEG pattern remains unclear. Furthermore, our analysis was limited to the presence of particular scalp topographies and not sequences of neural firings. Still, our data are in broad agreement with single-cell recordings in rodent models and neuroimaging studies in humans that have demonstrated the phenomena of offline task “reactivation” during rest and sleep. Note that the lack of significant change in microstate GEV between the two rest periods in the control group should not be taken to indicate that PVT performance has no impact on microstates since a PVT training session occurred prior to the baseline quiet rest period.

### There are two kinds of plastic changes in resting state EEG following learning

Here, we report the first use of scalp EEG to demonstrate the continuation of task-related activity patterns during post-training rest. This agrees with results reported by other investigators using other neuroimaging methods^10,44,45^. In addition, the inclusion of a control task suggests that these EEG changes are *task-specific*, or related to task- switching from the PVT to the VMT. Therefore, we distinguish between two types of EEG plasticity. First, we report localized increases in alpha and beta EEG power following learning that are unrelated to the power spectrum during training and unrelated to test performance. Studies of simulated driving indicate that increases in alpha and beta power are markers of fatigue^46^. We note that both driving simulation and our virtual maze task require extended use of mental systems for spatial navigation. Therefore, changes in the power spectrum may reflect the induction of fatigue, specifically in spatial navigation brain systems. In addition, we also report a second type of EEG change in which task-related spatial patterns persist into post-training rest, persist into subsequent sleep, and predict post-sleep change in performance on the VMT. Because of their substantially different characteristics and the fact that these changes are not correlated with each other, we propose that these two experience-dependent changes in the resting state EEG reflect separate, but co-occurring, neural processes. Thus, task training may produce multiple reverberations in the post-training resting state EEG. Because N2 occurs later in time than post-training rest, further work would be necessary to determine if any differences between rest and N2 were due to time elapsed, as opposed to behavioral state per se.

### The role of quiet waking in memory consolidation

The appearance of task-related EEG activity during rest could signify a role for quiet rest in memory consolidation. Indeed, it is increasingly clear that memory processing can occur during periods of quiet waking^47,48^. There are several reasons why waking rest might benefit memory. For one, the presentation of additional information can interfere with previously presented information, and hence it would be important to stabilize the original information as quickly as possible. For example, memory stabilization and consolidation of motor sequence learning can be disrupted by the presentation of a second sequence shortly after the original training^49^. When compared to active wake, periods of quiet waking can be relatively free of additional stimuli that might demand cognitive resources and induce learning, thereby disrupting the offline processing of previously acquired knowledge.

At the same time, mechanistically, the transition from active waking to quiet waking is accompanied by decreased acetylcholine and slowing of the EEG, producing a brain state during quiet rest that is more “sleep-like”^48,50^. Indeed, neural processes occurring during periods of quiet waking are similar to those that lead to memory consolidation during sleep. This is seen in of the appearance of hippocampal sharp-wave ripples and offline replay during quiet rest^51–53^.

Previous work indicates that brain activity during post-training quiet rest can potentiate memory consolidation^44,48,49,54^. Persistence of task-related patterns of hippocampal activity into post-encoding rest has previously been shown to be correlated with memory performance across waking^45^. However, the post-training resting state neural activity that is correlated with learning is not necessarily *the same* neural activity that occurs during task training. For example, slow oscillations (<1 Hz) during post-training rest are associated with improved memory in a declarative task while such oscillations are not thought to be associated with encoding^48^. In addition, sleep-dependent consolidation of a motor sequence is associated with increases in bilateral motor cortex connectivity, even though this connectivity did not significantly change during task training^54^. Here, we report a training-induced change in quiet waking brain activity that is negatively correlated with memory performance following sleep. We suggest that the neural activity reflected in this microstate topography during quiet rest following a spatial learning task not only correlates with, but possibly shapes, the extent to which the task will subsequently be consolidated during sleep. This is in agreement with previous work that has shown that resting state functional connectivity immediately after task learning predicts subsequent sleep-dependent memory consolidation^54^.

### M_TR_ predicts subsequent sleep dependent memory consolidation

Several models of sleep dependent memory processing propose that during wake the brain encodes memories and during sleep the brain consolidates them. It is hypothesized that as part of this process, memories must be actively tagged or marked for consolidation^54,55^. How exactly this proposed tagging might occur remains unclear and it is tempting to consider the possibility that the changes in M_TR_ that we report here are associated with the tagging process. However, the negative correlation between M_TR_ and sleep-dependent consolidation makes it is unlikely that M_TR_ is a direct EEG-correlate of the tagging of specific task-related memories. Future work is needed to clarify the relationship (if any) between recurrence of task-related EEG microstates and tagging.

There have been many attempts to identify functional correlates for the four canonical microstates^17^. The canonical microstate D, which closely resembles our M_TR_, has been linked to cognitive control, task-switching, and dorsal attention networks^56,57^. Therefore, our data suggest that training on the VMT may induce changes in activity within these networks that can be reactivated for several hours.

In any case, our results suggest that the relationship between task-related activity during resting states and memory may more complicated than generally believed, and future work is needed to elucidate the relationship between EEG microstate reactivation and memory consolidation.

## Conclusion

Here, we report the first observation of a task-related pattern in the scalp EEG that is also spontaneously present during subsequent rest and sleep. Although our data are not directly analogous to the “reactivation” of memory studied in rodents on the cellular level, these observations are broadly consistent with the hypothesis that post-learning resting states are a time during which recent experience continues to be processed “offline”. Furthermore, these data suggest that scalp-recorded EEG may offer a fruitful, low-cost method for future research exploring the effect of learning on resting-state brain function.
